# Chondrogenic differentiation of human bone marrow MSCs in osteochondral implants under kinematic mechanical load is dependent on the underlying osteo component

**DOI:** 10.3389/fbioe.2022.998774

**Published:** 2022-10-18

**Authors:** Graziana Monaco, Feras Qawasmi, Alicia J. El Haj, Nicolas R. Forsyth, Martin J. Stoddart

**Affiliations:** ^1^ AO Research Institute Davos, Davos, Switzerland; ^2^ Guy Hilton Research Centre, School of Pharmacy and Bioengineering, Keele University, Stoke-on-Trent, United Kingdom; ^3^ Hadassah Medical Center, Jerusalem, Israel; ^4^ Healthcare Technology Institute, Institute of Translational Medicine, University of Birmingham, Birmingham, United Kingdom

**Keywords:** osteochondral, chondrogenesis, crosstalk, tissue engineering, scaffold, signalling, mechanobiology

## Abstract

Chondrogenic models utilizing human mesenchymal stromal cells (hMSCs) are often simplistic, with a single cell type and the absence of mechanical stimulation. Considering the articulating joint as an organ it would be beneficial to include more complex stimulation. Within this study we applied clinically relevant kinematic load to biphasic constructs. In each case, the upper layer consisted of fibrin embedded hMSCs retained within an elastomeric polyurethane (PU) scaffold. These were randomly assigned to five base scaffolds, a cell-free fibrin PU base, viable bone, decellularized bone, 3D printed calcium phosphate or clinically used cement. This allowed the study of cross talk between viable bone and chondrogenically differentiating MSCs, while controlling for the change in stiffness of the base material. Data obtained showed that the bulk stiffness of the construct was not the defining factor in the response obtained, with viable and decellularized bone producing similar results to the softer PU base. However, the stiff synthetic materials led to reduced chondrogenesis and increased calcification in the upper MSC seeded layer. This demonstrates that the underlying base material must be considered when driving chondrogenesis of human cells using a clinically relevant loading protocol. It also indicates that the material used for bony reconstruction of osteochondral defects may influence subsequent chondrogenic potential.

## 1 Introduction

Articular cartilage overlays the articulating surfaces of bones providing a low friction gliding surface to allow free movement and acts to protect the underlying bone, preventing damage from the high forces generated within joints (Sophia Fox et al., 2009). Cartilage defects are increasingly common and can be divided into three major classes: partial thickness defects, full thickness defects and osteochondral defects depending on the depth of the damage (Nukavarapu and Dorcemus, 2013). Cartilage defects present a unique clinical challenge due to their poor self-healing capacity, largely due to its avascular nature that impedes access of blood and bone marrow mesenchymal stem/stromal cells (MSCs) from the surrounding environment (Hunziker and Rosenberg, 1996; Buckwalter and Mankin, 1998). The crosstalk between the cartilage, underlying bone and any regenerating tissue will play a role on the resultant *de novo* tissue (Gomoll et al., 2010; Madry, 2010). Yet *in vitro* model systems generally lack this critical interaction, furthermore they also typically lack mechanical stimulation, a key aspect of cartilage biology. In many clinical trials of symptomatic cartilage defects, a modified autologous chondrocyte implantation (ACI) technique has been used with success by replacing the autologous chondrocytes with hMSCs seeded into collagen scaffolds (Wakitani et al., 2002; Wakitani et al., 2004; Kuroda et al., 2007). The substitution of chondrocytes with MSCs is also increasingly common as bone marrow aspirate harvest is relatively simple to obtain in comparison to cartilage biopsy required for chondrocyte expansion. MSCs as an alternative cell source for cartilage repair are relatively easy to isolate from a variety of tissues and represent an emerging ATMP treatment (Schneider et al., 2010; Bieback et al., 2011). However, in general current surgical treatments do not ensure consistent regeneration of hyaline cartilage, leading predominantly to fibrous tissue and hence remain controversial (Knutsen et al., 2007; Armiento et al., 2018). Therefore, there is a critical need to better study and understand healing mechanisms to achieve more effective therapies for cartilage regeneration through the development of new tissue engineering approaches. For this, to be successful, improved *in vitro* culture models will be required.

One of the major issues associated with *in vitro* MSCs chondrogenesis for cartilage therapy is their progression towards terminal differentiation and hypertrophy (Pelttari et al., 2006; Mueller and Tuan, 2008). It has been speculated that the rapid progression of MSCs towards hypertrophy during chondrogenic induction *in vitro* by using pellet culture, is caused by the loss of the important spatial and temporal signalling networks that exist for *in vivo* chondrogenically differentiating populations of cells (Pelttari et al., 2006). Accordingly, the interaction between cartilage and the subchondral bone is increasingly a focus of preclinical and clinical research (Gomoll et al., 2010; Madry et al., 2016). As such, the crosstalk and the organization of the cells within the tissue is essential for the tissue’s normal development, homeostasis and repair. The ability to manage and reproduce the complex osteochondral unit from a cell point of view is still challenging but represents one of the key factors for a successful tissue regeneration. Since most tissues in the body, including osteochondral tissue, consist of more than one cell type, the development of a suitable co-culture system becomes an important requirement to finally achieve a functional cartilage regeneration both for clinical needs and for research purposes. Attempts have been made to establish osteochondral models.

Therefore, the aim of this study was the development of a more complex hybrid *in vitro*—*ex vivo* osteochondral model composed of an *ex vivo* bone explant cylinder underlying the *in vitro de novo* cartilage zone, represented by a fibrin poly (ester-urethane) (PU) construct asymmetrically seeded with hMSCs (Gardner et al., 2017a). This study primarily aimed to investigate the effect of bone and its putative biological and mechanical signals on the chondrogenic differentiation of human bone marrow MSC residing in the upper PU: fibrin scaffold. In parallel, the clinically relevant materials calcium phosphate and bone cement were used as a stiff osteochondral base in place of bone. In addition, the effect of joint-simulating mechanical load has been demonstrated to be beneficial for hMSC chondrogenesis (Li et al., 2010a). Therefore, the effect of mechanical loading was assessed, concomitantly with the presence of the bone and with its alternatives bases, on hMSC chondrogenesis seeded within the upper PU:fibrin scaffold. The aim of this study was to investigate if *ex vivo* bone affects the chondrogenic differentiation of hMSCs within a bilayer scaffold. We hypothesized that soluble signals from live bone would change the phenotype of the MSCs differentiating in the upper layer. Furthermore, we hypothesized that changing the 2 mm base material to a rigid material would modify the response to load compared to the soft PU base.

## 2 Materials and methods

### 2.1 Donor information

Human bone marrow aspirate was obtained with full ethical approval (KEK-ZH-NR: 2010-0444/0) and MSCs were isolated using standard methods (Gardner et al., 2015). See Table 1 for donor details. In brief, after Ficoll gradient separation mononuclear cells were seeded at a density of 50,000 cells/cm2 into culture flasks and left to attach for 96 h in αMEM supplemented with 10% FBS, 5 ng/ml basic fibroblast growth factor (bFGF) (Peprotech, Rocky Hill, United States) and 100 U/ml penicillin and 100 μg/ml streptomycin (Gibco, Carlsbad, United States). The medium was changed after 4 days and the attached cells were allowed to grow to 80% confluence before passaging. Expanded cells were seeded at density of 3,000 cells/cm2 into fresh culture flasks and cultured in complete expansion medium.

**TABLE 1:** Human sequences of self-designed oligonucleotide primers and probes used for real-time PCR analysis.

Donor	Donor site	Age (y)	Gender
Donor 1	vertebra (TH12/L2)	66	Female
Donor 2	Vertebra	22	Male
Donor 3	vertebra (TH7/9)	69	Male

### 2.2 Viable bone cylinder preparation from bovine knee joints

Bovine knee joints were collected from 6-month-old calves obtained from a local abattoir (Metzgerei Angst AG, Zurich, CH) within 48 h of slaughter. Drilling from the cartilage femur surface was performed by using the Bosch PBD 40 compact drilling machine supported by saline irrigation to constantly keep a hydrated environment during drilling. Osteochondral explants cylinders of 8 mm diameters were produced using a diamond coated custom-made trephine drill (Peertools AG, Ftan, CH). The cylindrical explants were washed with 1000 U/mL penicillin and 1,000 μg/ml streptomycin for 15 min. Then, the explants were further cut with an Exakt 300 band circular saw (Exakt Apparatebau GmbH & Co.KG, DE) to achieve cancellous bone cylinders with a final thickness of 2 mm and a diameter of 8 mm. These cylinders were soaked for 15 min in 1,000 U/ml penicillin and 1,000 μg/ml streptomycin and then for other 15 min in 100 U/ml penicillin and 100 μg/ml streptomycin. Cancellous bone cylinders were then transferred into 24 well plates and cultured in Dulbecco’s modified eagle medium (DMEM-HG, 4.5 g/L-glucose; Gibco) supplemented with 10% fetal bovine serum (FBS, Gibco) and 100 U/ml penicillin and 100 μg/ml streptomycin, at 37°C and 5% CO_2_ until the assembling with the upper MSC seeded PU-scaffold.

### 2.3 Decellularization of bone cylinders

Bone cylinders were exposed to five freezing-thawing cycles alternating between liquid nitrogen for five minutes and a 56°C water bath for five minutes (Jahn et al., 2010).

### 2.4 Seeding of fibrin-poly (ester-urethane) scaffolds

Elastomeric polyurethane (PU) scaffolds (2 mm height × 8 mm diameter) were created by salt leech synthesis. Scaffolds were asymmetrically seeded to better represent the superficial zone found in articular cartilage (Gardner et al., 2017a). Monolayer expanded MSCs (3 × 106 cells per scaffold) were resuspended in 37.5 µl of 33 mg/ml fibrinogen (Baxter, Vienna, Austria). The fibrin cell mixture was then rapidly mixed in the presence of the PU scaffold with an equal volume of 1 unit/ml thrombin (Baxter, Vienna, Austria). After 1 h at 37°C a further 500,000 cells were added to the surface of the scaffold and left to adhere. Scaffolds were cultured in a medium consisting of: Dulbecco’s modified Eagle medium (4.5 g/L glucose (Gibco, Carlsbad, United States), 0.11 g/L sodium pyruvate, 50 μg/ml L-ascorbic acid 2-phosphate sesquimagnesium salt hydrate, 1 × 10-7 M dexamethasone, ITS + premix containing insulin 6.25 μg/ml, transferrin 6.25 μg/ml and selenious acid 6.25 ng/ml, bovine serum albumin 1.25 mg/ml and linoleic acid 5.35 μg/ml (Corning, Bedford, United States), 1% (v/v) Non-essential amino acids (Gibco, Carlsbad, United States), 100 U/mL penicillin and 100 μg/ml streptomycin (Gibco, Carlsbad, United States) and 5 μM 6-aminocaproic acid to reduce fibrin degradation (Kupcsik et al., 2009).

### 2.5 Preparation of 3D printed ceramic base

Osteoink calcium phosphate cement ink was use to 3D print the ceramic base as recommended by the manufacturer (Osteoink^TM^, RegenHU, Villaz-St-Pierre, Switzerland). The ink is similar to the CE-marked ink developed by Innotere GmbH (Germany) and is mainly composed of α-tricalcium phosphate (α-TCP) with smaller amounts of hydroxyapatite, monetite, calcite and β-TCP, and a carrier liquid composed of triglycerides and surfactants (Heinemann et al., 2013). Setting of the cement occurs *via* α-TCP hydrolysis.

### 2.6 Preparation of the cement base

Acrylic casting resin SCS-Beracryl D-28 (Suter Kunststoffe AG, Fraubrunnen, Switzerland) was used to prepare the solid cement base as per manufactures instructions. In brief, the resin acrylic SCS-Beracryl D-28 and hardener was mixed in approximately equal weight proportions and mixed well. After casting, the samples were left to harden for 1 h.

### 2.7 Preparation of osteochondral combinations

**FIGURE 1:**
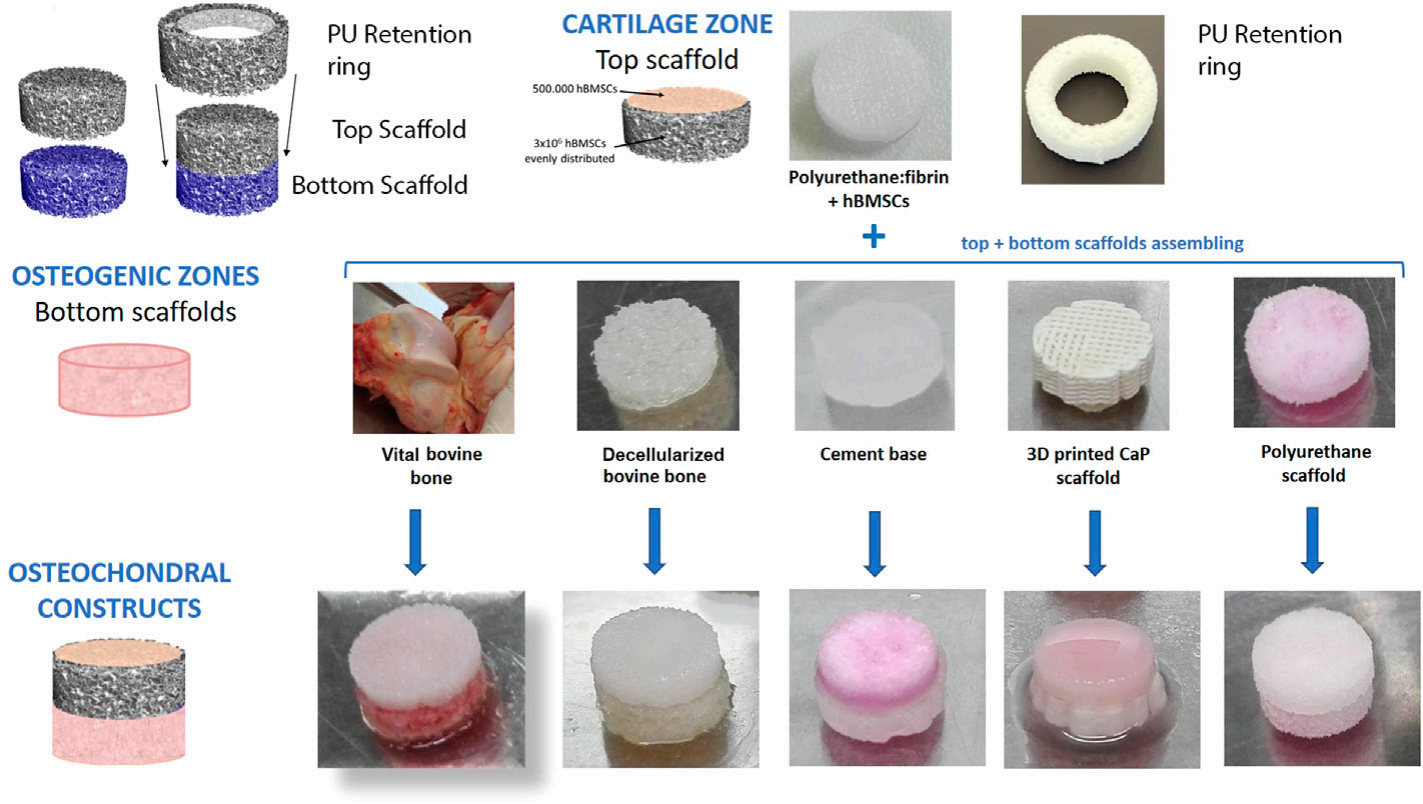
Overview of experimental design.

hMSC seeded scaffolds were placed over the relevant base material and held together using a PU ring produced in the same manner as the upper PU scaffold. For an overview of the experimental Design, see Figure 1.

### 2.8 Application of multi-axial mechanical load

Osteochondral constructs were loaded using a custom-made multiaxial load bioreactor, designed to apply both dynamic shear and compressive loading, based on a tribological system analysis of natural articulating joints (Wimmer et al., 2004). Loaded scaffolds were exposed to 10% compression superimposed on top of a 10% pre-strain and shear loading (± 25°C) at 1 Hz for one hour a day five times a week over 7 and 28 days. 10% compression and 25°C rotation were chosen based on results from previous work (Li et al., 2010a). Load cells, located underneath the sample holders, were used to detect contact between the ceramic ball and scaffold in order apply 10% pre-compression before the initiation of 10% cyclic compression. Loading was carried out in an incubator at 37°C, 5% CO_2_ and 80% humidity. Control scaffolds were kept in free-swelling culture conditions and harvested in parallel at the same timepoints of the loaded samples. The culture media was changed three times per week during the loading period.

### 2.9 Real Time PCR

RNA was isolated using TRI reagent and cDNA synthesized using Superscript VILO cDNA synthesis kit (Thermo Fisher) as per manufacturers recommendations. Either custom primers (Microsynth, Balgach, Switzerland) or Gene Expression Assays (Applied Biosystems, Carlsbad, United States) (Table 2) were used. Real Time PCR was performed using TaqMan^®^ Master Mix and an Applied Biosystems QuantStudio 6 Flex Real-Time PCR System (Applied Biosystems, Carlsbad, United States. Data was analysed using the 2-ΔΔCt method to day 0 with human RPLP0 as the endogenous control.

**TABLE 2:** Details of source material from each donor used.

Gene	Primer forward (5′-3′)	Primer reverse (5′-3′)	Probe (5′ FAM- 3′ TAMRA)
COL2A1	5′-GGC AAT AGC AGG TTC ACG TAC A-3′	5′-GAT AAC AGT CTT GCC CCA CTT ACC-3′	5′-CCT GAA GGA TGG CTG CAC GAA ACA TAC-3′
COL10A1	5′-ACG CTG AAC GAT ACC AAA TG-3′	5′-TGC TAT ACC TTT ACT CTT TAT GGT GTA-3′	5′-ACT ACC CAA CAC CAA GAC ACA GTT CTT CAT TCC-3′
ACAN	5′-AGT CCT CAA GCC TCC TGT ACT CA-3′	5′-CGG GAA GTG GCG GTA ACA-3′	5′-CCG GAA TGG AAA CGT GAA TCA GAA TCA ACT-3′
RunX2	5′-AGC AAG GTT CAA CGA TCT GAG AT-3′	5′-TTT GTG AAG ACG GTT ATG GTC AA-3′	5′-TGA AAC TCT TGC CTC GTC CAC TCC G-3′
OC	5′-AAG AGA CCC AGG CGC TAC CT-3′	5′-AAC TCG TCA CAG TCC GGA TTG-3′	5′-ATG GCT GGG AGC CCC AGT CCC-3′
RPLP0	5′-TGG GCA AGA ACA CCA TGA TG-3′	5′-CGG ATA TGA GGC AGC AGT TTC-3′	5′-AGG GCA CCT GGA AAA CAA CCC AGC-3′
COMP	Applied Biosystems TaqMan Gene Expression Assay Hs00164359_m1		
PRG 4	Applied Biosystems TaqMan Gene Expression Assay Hs00981633_m1		
Sox9	Applied Biosystems TaqMan Gene Expression Assay Hs00165814_m1		
ALP	Applied Biosystems TaqMan Gene Expression Assay Hs00758162_m1		

RPLP0, ribosomal protein large P0 housekeeping gene; COL2A1, collagen type 2; COL10A1, collagen type 10; ACAN, aggrecan; RunX2, runt-related transcription factor 2; OC, osteocalcin; COMP, cartilage oligomeric protein; PRG, 4, Proteoglycan 4. Applied Biosystems TaqMan Human Assays on demand used for qRT-PCR., Sox9, SRY (sex determining region Y)-box 9 cartilage transcription factor; ALP, alkaline phosphatase.

### 2.10 Biochemical analysis

For biochemical analysis, one half scaffold was digested in 0.5 mg/ml Proteinase K (Roche, Basel, Switzerland) at 56°C for 16 h. Proteinase K (PK) was then deactivated with a 10 min incubation at 96°C, and the samples stored at -20°C for analysis. Total amount of glycosaminoglycans (GAGs) produced by cells, measured in both collected media and PK digests, was determined using the 1.9-dimethyl methylene blue (DMMB) assay and normalised to the DNA content of each PK digest measured using Hoechst 33,258 dye (Polysciences Inc., Warrington, United States).

### 2.11 Bone sectioning and viability assessment of osteocytes using lactate dehydrogenase activity staining

Bone cylinders were cut by using Leica annular saw (Leica AG, Glattbrugg,CH) to achieve sections of 250 µm–300 µm. Lactate dehydrogenase (LDH) staining solution (5% polypeptide base solution, 60 mM lactic acid (final conc. 5.4 mg/ml), 17.5 mg Nicotinamide adenine dinucleotide (NAD final conc. 1.75 mg/ml) was prepared (Jahn and Stoddart, 2011). The pH was brought to 8.00 and 30 mg Nitroblue Tetrazolium (NBT, Sigma N5514) was then added. The bone sections were washed in PBS and placed in separate well where 500 µl of freshly prepared LDH staining solution was added. The sections were incubated at 37°C for 4 h in the dark, washed with warm (50°C–56°C) distilled water and then fixed with 4% paraformaldehyde at 4°C for 10 min. After a brief wash in deionized water, the sections were placed on glass slides and mounted with water based mountant (hydromount DAKO, National Diagnostics, HS-106).

### 2.12 Histological analysis

Methanol fixed PU/fibrin/MSC samples were frozen in OCT compound (R. Jung GmbH, Nussloch, Germany) before being sectioned (12 µm thick) on a cryotome (Carl Zeiss AG, Oberkochen, Germany) and adhered to Superfrost Plus slides (Thermo Fischer scientific, Waltham, United States). Slides were stored at −20°C.

### 2.13 Safranin O/Fast green staining

OCT compound was removed from the cryosections by rinsing in distilled water for ten minutes prior to Safranin O staining. This was followed by a twelve minute incubation in Weigert’s Haematoxylin. The slides were then placed in lukewarm tap water for ten minutes, briefly washed in distilled water and then placed in 0.02% (v/v) fast green in 0.01% (v/v) acetic acid in deionised water for five minutes. Fast green staining was followed by thirty seconds in 1% (v/v) acetic acid and then five minutes in 0.1% (w/v) Safranin O solution (Chroma-Gesellschaft Schmid GmbH & Co., Münster, Germany). The slides were then dehydrated by immersing them in 96% ethanol twice (one minute each) and then 100% ethanol twice (two minutes each). Slides were then placed in 100% xylene for two minutes twice before being mounted using Eukitt mounting medium.

### 2.14 Von Kossa staining

OCT compound was removed from the cryosections by rinsing in distilled water for ten minutes prior to Von Kossa staining. This was followed by a thirty-minute incubation in 5% (v/v) Silver Nitrate (Sigma Aldrich, Sigma-Aldrich, Buchs, Switzerland) and parallel exposure to strong light. The slides were then rinsed three times ten minutes with deionized water and then placed in 5% (v/v) Sodium Thiosulfate (Sigma Aldrich, Sigma-Aldrich, Buchs, Switzerland) for ten minutes to fix the previous staining. The sections were washed again three times ten minutes with deionized water and counterstained per 10 min with 0.1% (v/v) Nuclear fast red (Fluka, St. Louis, United States). The slides were then washed as described in the previous steps and dehydrated by immersing them in 70% ethanol per ten seconds, 96% ethanol per one minute and then 100% ethanol twice (two minutes each). Slides were then placed in 100% xylene for two minutes twice before being mounted using Eukitt mounting medium.

### 2.15 Statistical analysis

Data was analysed using 2-way ANOVA with Tukey multiple comparison, a *p* value > 0.05 was considered significant.

## 3 Results

### 3.1 Gene expression profiles of hMSCs seeded into fibrin-poly (ester-urethane) scaffolds after seven and twenty-eight days of culture

**FIGURE 2:**
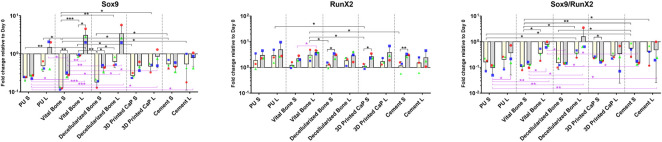
Gene expression of transcription factors SOX9 and RUNX2 in various scaffold systems under static (S) and loaded (L) conditions. Load generally led to an increase in both transcription factors. The load induced increase in SOX9 was most pronounced in the PU, vital bone, and bone dead groups. When considering the SOX9/RUNX2 ratio, the higher values were observed in the loaded groups with the bony bases. *p < 0.05, **p < 0.01, ***p < 0.001. Black significance bars designate differences on Day 7 among all groups or day 7 vs. day 28 within the same group. Purple significance bars designate differences on Day 28.

**FIGURE 3:**
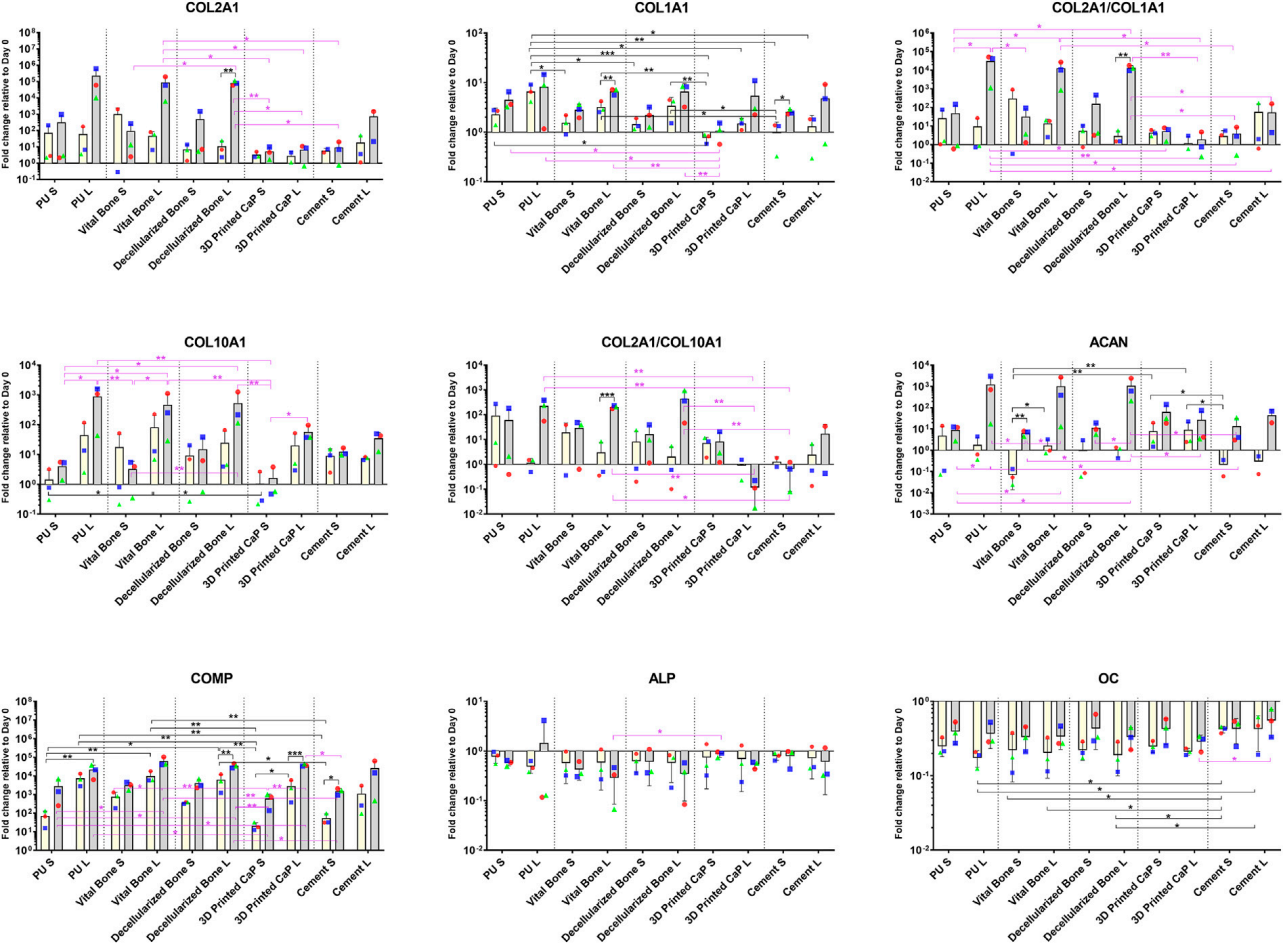
Gene expression of cartilage (COLA2A1, COMP, ACAN), hypertrophy (COL10A1) and bone (COL1A1, ALP, OC) related genes. Load generally led to an increase in all markers except ALP. Load combined with the PU, vital bone, and bone dead groups led to particularly favourable ACAN expression and improved COL2A1/COL1A1, COL2A1/COL10A1 ratios. *p < 0.05, **p < 0.01, ***p < 0.001. Black significance bars designate differences on Day 7 among all groups or day 7 vs. day 28 within the same group. Purple significance bars designate differences on Day 28.

After 7 and 28 days of culture the gene expression profile of the MSCs in the upper scaffold was assessed (Figures 2, 3). Both transcription factors studied, SOX9 and RUNX2 were upregulated by mechanical load. The increase in Sox9 expression was most pronounced in the PU, viable bone and decellularized bone groups whereby day 28 expression had increased above day 0 levels. This led to a noticeable improvement in the SOX9/RUNX2 ratio, an indicator of chondrogenesis, in these groups (Figure 2).

Load also increased aggrecan and collagen 2 expression in the PU, viable bone and decellularized bone groups (Figure 3). While printed CaP and Cement based groups had a lower expression by day 28 and no noticeable response to load. By day 28, load induced a significant increase in collagen 10, which was most noticeable in the PU, viable bone and decellularized bone groups. The Col 2/Col 10 ratio demonstrates a significant benefit of load in the PU, viable bone and decellularized bone groups over time, the Col 2/Col 10 ratio decreased in the printed CaP group, indicating an unfavourable condition.

Investigating other pathways, Collagen 1 showed an increased expression, which was similar in all groups by day 28 (Figure 3). However, the PU, viable bone and decellularized bone groups also had a noticeable increased expression by day 7. ALP expression generally decreased with load, however few changes were significant. The expression of osteocalcin was generally higher with load in all groups, with no obvious differences between the groups. COMP expression levels generally increased over time in all groups and this was more pronounced with all loaded groups.

Taken together, the expression of cartilage related markers was increased by load and formed two clusters of expression. The PU, viable bone and decellularized bone groups showed a beneficial profile that was similar between the groups, whereas CaP and cement displayed a poorer chondrogenesis. The more bone related markers Col1, ALP, osteocalcin and Runx2 showed no specific base related trends.

### 3.2 Quantification of DNA content and sulphated glycosaminoglycan in fibrin-poly (ester-urethane) scaffolds and released into culture media

**FIGURE 4:**
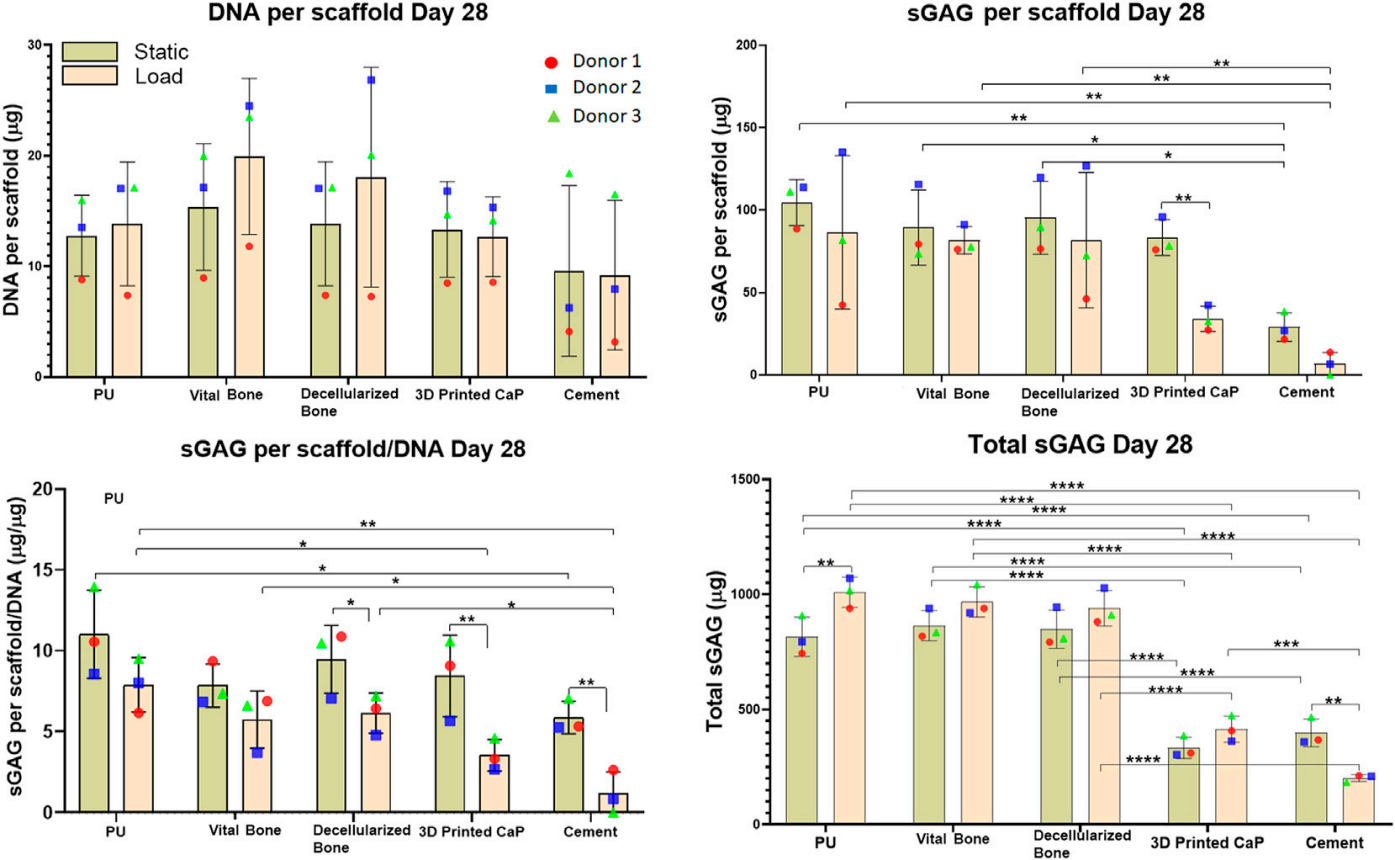
DNA, GAG per scaffold, Total GAG per scaffold/DNA, Total sGAG released into the media and total sGAG (media plus scaffold) produced over the entire 28-days. Load led to reduced sGAG per scaffold/DNA, mainly due to the load leading to an increased release of GAG into the medium. However, with the exception of the cement group, load led to an increase in total sGAG produced. The total sGAG produced was similar for PU, viable bone and decellularized bone, while the use of a 3D printed or cement base led to a significant reduction of total sGAG.

Under static conditions all groups showed a similar DNA content after 28 days of culture (Figure 4). This was largely unaffected by load, except for the vital bone and bone dead groups where a slight increase in DNA content was observed under loading conditions compared with static.

sGAG deposited in the scaffold: After 28 days of MSC chondrogenesis, no significant differences were observed in the sGAG deposition in PU, vital bone and bone dead groups (Figure 4). However, load led to a significant decrease (**p < 0.01) of sGAG deposition in the 3D printed CaP group. A similar but not significant decrease was observed for the cement group. However, the sGAG deposition within the Cement group both under static and load conditions was significantly lower than the corresponding static or loaded vital bone (**p < 0.01 and *p < 0.05), bone dead (**p < 0.01 and *p < 0.05) and PU (**p < 0.01) groups.

GAG per scaffold/DNA ratio at day 28: the scaffold sGAG/DNA ratio was investigated at day 28 to evaluate if the sGAG incorporated in the scaffold was due to higher MSC proliferation than differentiation (Figure 4). The scaffold sGAG/DNA ratio showed a similar trend observed for the GAG per scaffold. An overall reduction of the scaffold GAG/DNA ratio under loading was consistent among all the groups and was significantly more pronounced for bone dead (*p < 0.05), 3D printed CaP (**p < 0.01) and cement groups (**p < 0.01). Under static conditions, PU led to the highest GAG/DNA level and this reached significance when compared with cement static (*p < 0.05), with a tendency to higher GAG/DNA ratio when compared with the vital bone, 3D Printed CaP and cement loaded groups.

Within the loaded groups comparison, PU showed again the best scaffold sGAG/DNA ratio that was significantly higher compared with 3D Printed CaP (**p < 0.01) and Cement (*p < 0.05) groups. Cement loaded group showed the worst scaffold sGAG/DNA ratio significantly lower compared with PU (**p < 0.01), bone dead (*p < 0.05) and vital bone (*p < 0.05) loaded. In addition, the cement load scaffold sGAG/DNA was a notably lower also in comparison with all the other static groups. 3D Printed CaP loaded group showed notably lower GAG/DNA then bone dead and PU static groups.

**FIGURE 5:**
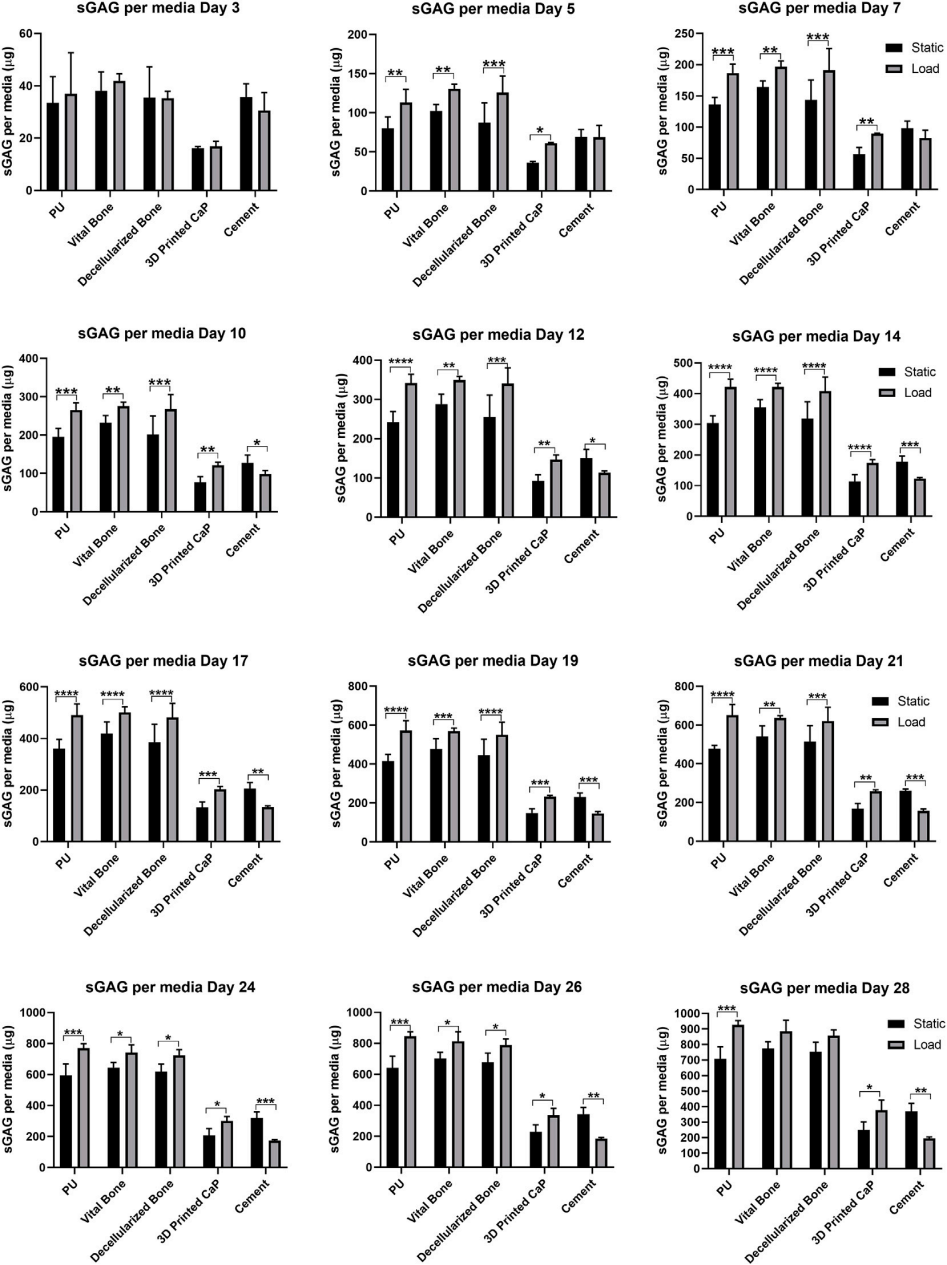
sGAG released into media by day. Media was sampled every two days and the sGAG contained within was quantified. By day 5 significant load induced differences were observed due to load and these were maintained throughout the experiment. *p < 0.05, **p < 0.01, ***p < 0.001, ****p < 0.0001.

When using the PU: fibrin system, a large portion of the sGAG produced is released into the culture media. Therefore, GAG per media was also monitored and analyzed at each media change in order to investigate the trend of GAG release over 28 days of hMSCs chondrogenic differentiation (Figure 5). Over 28 days, an overall increase of sGAG per media was observed under loading conditions compared with comparable static conditions, with 3D Printed CaP (*p < 0.05) and PU (***p < 0.001) groups reaching significance. The cement base was the only group where a significant decrease (**p < 0.01) was detected. Notably, the 3D Printed CaP and cement groups had a lower GAG production compared with the other three groups. Among the static groups, the highest media sGAG level was detected for vital bone group while among the loaded groups the highest media sGAG level was detected for PU. However, we did not find significant differences between PU, vital bone and bone dead groups by comparing either static or loaded conditions. Differently, PU (****p < 0.0001), vital bone (****p < 0.0001) and bone dead (****p < 0.0001) groups released a significantly higher sGAG into the culture media compared with 3D Printed CaP and Cement either under static or under loading conditions. In addition, 3D Printed CaP loaded group showed significantly higher media sGAG (**p < 0.01) compared with cement loaded group. No significant differences occurred under static conditions between 3D Printed CaP and cement even though 3D Printed CaP produced and released in the culture media more sGAG than the cement group.

### 3.3 Histology

**FIGURE 6:**
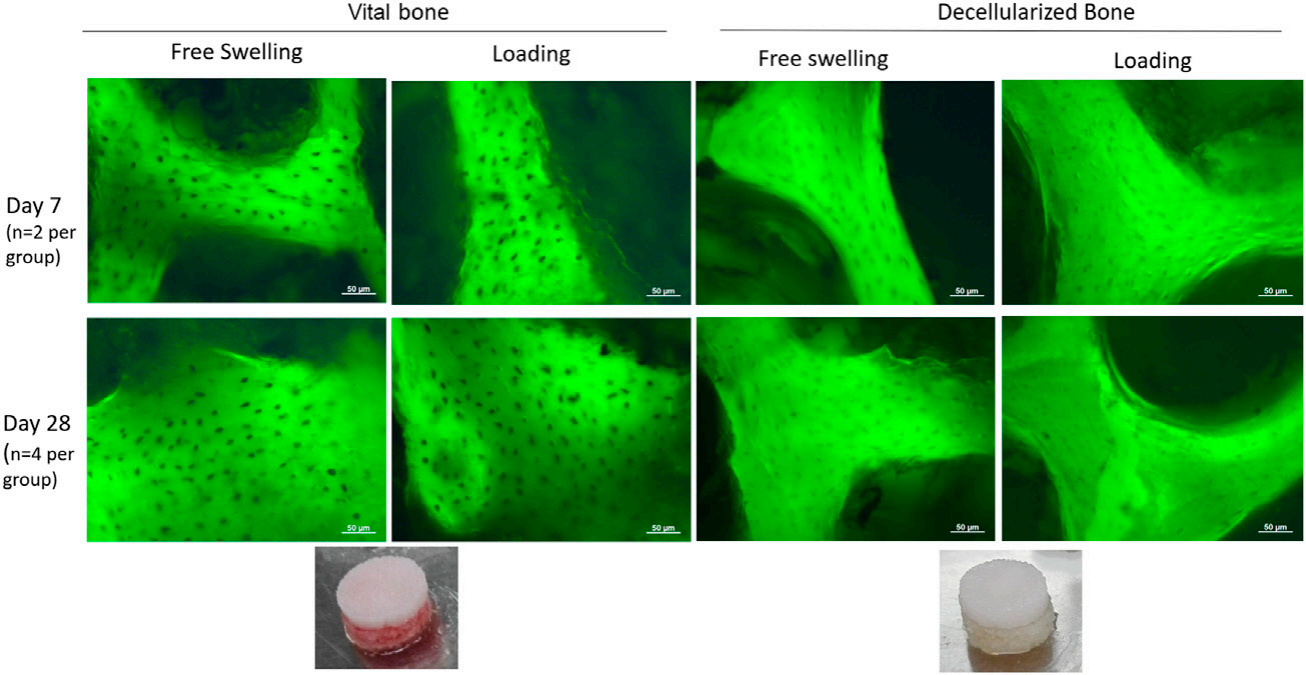
LDH staining of viable osteocytes within cultured bone explants. Dark spots indicate a viable osteocyte visible against the autofluorescent bone. Osteocytes in viable bone remained viable for up to 28 days, while the decellularization process was successful at removing all viable osteocytes.

In order to investigate the effect of the bone, the assessment of its viability over all the culture time was ensured by performing the lactate dehydrogenase viability staining at different timepoints. Viable bone was observed over the full 28 days, while the lack of LDH stained lacunae demonstrated the successful decellularization process (Figure 6).

**FIGURE 7:**
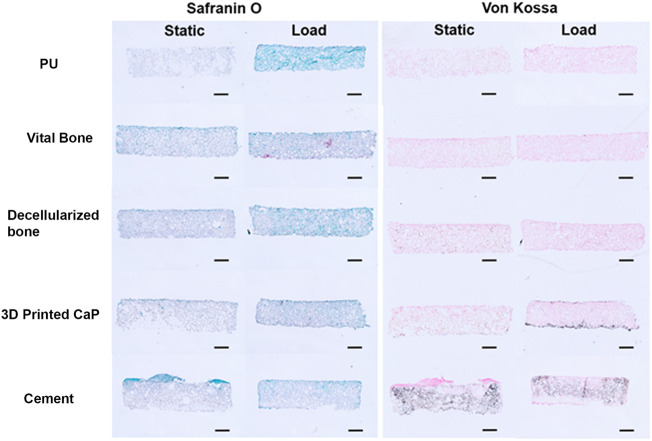
Example Safranin O and Von Kossa histology after 28 days of culture. Safranin O staining was minor due to the high resale of produced GAG into the culture medium. Von Kossa staining was observed at the interface (3D Printed CaP) or throughout (cement) the upper MSC laden scaffold. This was not observed when PU or bone was used as the base. (Scale bar = 1 mm).

Due to the large release of GAG into the medium, little safranin O-stained GAG matrix was observed in the upper scaffold after 28 days (Figure 7). However, von Kossa staining demonstrated considerable differences between the groups (Figure 7). While there was nonspecific staining of the cell free polyurethane base, there was no calcification observed in the upper cell seeded PU scaffold in any of the donors for the PU, viable bone and decellularized bone groups. However, with the exception of donor 3, there was significant von Kossa staining in the 3D printed calcium phosphate and cement groups. The 3D printed calcium phosphate demonstrated more calcification at the lower edge of the scaffold at the interface with the base, while the von Kossa staining was more evenly distributed throughout the scaffold in the cement group.

## 4 Discussion

Integration of kinematic load into *ex vivo* osteochondral culture models would allow a closer representation of the *in vivo* environment (Vainieri et al., 2018). This strategy could provide a new model to study healing or the regenerative processes of articular cartilage in a more joint-like environment, especially when various mechanical loading patterns can be applied to the model. In recent years, there has been a considerable effort to produce bioreactors and loading devices (Heath and Magari, 1996; Li et al., 2004; Lee et al., 2017; Meinert et al., 2017; Daly et al., 2018; Jonnalagadda et al., 2018; Nazempour and Van Wie, 2018). The bioreactors might be a supporting tool to expose cells seeded in a scaffold structure or *ex-vivo* explants, to different forms of mechanical load. This can be used to either develop tissue engineered implants or to better study the effect of mechanical load on tissue healing by simulating and predicting *in vivo* processes (Grad et al., 2011). It is quite challenging to achieve and faithfully reproduce complex *in vivo* load, as the motion pattern can vary greatly even within the same joint (Grad et al., 2011). Nevertheless, even rudimentary mechanical stimulation is highly desirable. Within this study we applied compression and shear mechanical load to various osteochondral implant composites. The primary aim was to assess effect of load and the potential crosstalk between viable bone and MSC laden constructs. To control for the mechanical environment, decellularized bone, calcium phosphate printed base, and a cement base were used. The devitalized bone also offered a control to the viable bone soluble molecules produced by the viable cells. When considering the two main hypothesis, in the current study neither were proven correct.

We hypothesized that soluble signals from live bone would change the phenotype of the MSCs differentiating in the upper layer. When considering the two main experimental groups required for this analysis, viable bone base and decellularized bone base, no differences at the gene expression level could be detected. Other studies have demonstrated a soluble cross talk between osteoblasts, chondrocytes and MSCs (Funck-Brentano and Cohen-Solal, 2011; de Vries-van Melle et al., 2014a; Leyh et al., 2014; Glueck et al., 2015). There are reasons why this may not have been observed in this study. Potentially, biologically active molecules were leaching out of the decellularized bone driving a similar response to live bone. Alternatively, the distance between the bone and the MSCs in the upper scaffold may have been too great, with the soluble signals only affecting the MSCs in the lowest part of the scaffold. These effects may have been diluted out when using a destructive PCR analysis and suggest more spatial phenotyping would be required. Furthermore, studies which have shown the most dramatic effects utilized an osteochondral defect model (de Vries-van Melle et al., 2014a; de Vries-van Melle et al., 2014b) where the implanted material would have a more confined environment. While it is also possible that soluble molecules from bovine bone are not able to adequately stimulate human cells, an effect of bovine subchondral bone on human bone marrow MSCs has previously been observed (de Vries-van Melle et al., 2014a) suggesting the lack of signalling arises elsewhere. Therefore, an enclosed osteochondral defect model such as that developed by Vaineri et al. would lead to more reliable data (Vainieri et al., 2018). While no changes were observed with the PCR data, differences were seen at the histological level, particularly in the Von Kossa staining pattern. There is also the possibility that the total cell density is not sufficient, and the load induced fluid flow in this unconfirmed environment led to a washout of the secretome rather than a cross talk.

Furthermore, we hypothesized that changing the 2 mm base material to a rigid material would modify the response to load compared to the soft PU base. This also was not the case, as both the live and decellularized bone bases had similar responses to the softer PU base. This suggests there is a critical threshold above which the load response occurs and this is already reached when using the PU: fibrin scaffolds. As it has been previously shown that shear superimposed on compression leads to the mechanical activation of TGF β, this may be one of the key motion patterns required to drive the response (Gardner et al., 2017b). As frequency and amplitude has been shown to modify the chondrogenic response (Li et al., 2010b) it suggests the mechanical activation occurs in the liquid interface during the shear application, and then compression drives the activated TGF β protein into the scaffold. Once the bulk properties of the construct reach a threshold to allow this to occur, stiffening the construct further would not necessarily lead to increased protein activation.

Unexpectedly, the printed CaP and cement base groups led to a detrimental effect on the MSC chondrogenesis. Collagen 2 and aggrecan expression was decreased in comparison to the other three groups, and GAG production was reduced. No obvious difference in DNA content was observed between the groups, suggestion viability was not a reason for the reduced chondrogenesis. In both cases, an increase in calcium deposition was observed in the upper MSC laden scaffold, suggesting a more bony phenotype was induced. This was not apparent in the PCR data and we have not yet identified the underlying pathway involved. The exact mechanism may be different for the two materials, as they have different porosity and calcium content, which may affect nutrient availability and final medium composition respectively. The negative effect for the cement and printed CaP groups was most noticeable with reduced GAG production, and lower collagen 2 mRNA expression levels, these aspects need investigating further. This result may have a clinical significance as both of these materials are used clinically, and this data suggests detrimental effects on the developing cartilage when compared to natural bone graft. In the case of the 3D printed calcium phosphate base, it is possible that the material is affecting media calcium and phosphate levels. A study using a similar material from Innotere GmbH, Germany, demonstrated that 3D printed calcium phosphate materials decreased media calcium levels, while phosphorus levels were initially increased over the first 14 days (Kilian et al., 2020). In that study, the base material led to improved GAG and Collagen II production when using human chondrocytes. Therefore, it is possible the biological outcome is cell specific, but the influence of the base material should not be overlooked. The response to load is also likely to be cell specific. The negative effects seen could also be related to an induction of apoptosis and future studies should study this in more detail.

Therefore, we can conclude that within ten different conditions it is possible to categorize the ten experimental groups in two groups: PU, vital bone and bone dead belong to the group that showed similar chondrogenic potential that was increased by load, while 3D Printed CaP and cement groups produced and released less sGAG and were less responsive to loading.

In summary, the response of load was dependent on the material used as the subchondral bone component, with PU, viable bone and decellularized bone showing similar profiles at the gene and protein level. The maintenance of viable osteocytes of 28 days shows the feasibility of this coculture approach and furthers studies will be performed to assess more regionalized differences. Data within this study suggests care should be taken when using printed calcium phosphate or cement as a filler for osteochondral defects as the chondrogenic response may be inhibited compared with natural bone.

## Data Availability

The raw data supporting the conclusions of this article will be made available by the authors, without undue reservation.
